# Pyroptosis-Related lncRNAs Predict the Prognosis and Immune Response in Patients With Breast Cancer

**DOI:** 10.3389/fgene.2021.792106

**Published:** 2022-03-14

**Authors:** Xia Yang, Xin Weng, Yajie Yang, ZhiNong Jiang

**Affiliations:** ^1^ Department of Pathology, Sir Run Run Shaw Hospital, School of Medicine, Zhejiang University, Hangzhou, China; ^2^ Department of Pathology, Shenzhen Second People’s Hospital, Shenzhen, China

**Keywords:** breast cancer, pyroptosis, lncRNA, prognosis, immune response

## Abstract

**Background:** Breast cancer (BC) is the most common malignant tumor and the leading cause of cancer-related death in women worldwide. Pyroptosis and long noncoding RNAs (lncRNAs) have been demonstrated to play vital roles in the tumorigenesis and development of BC. However, the clinical significance of pyroptosis-related lncRNAs in BC remains unclear.

**Methods**: Using the mRNA and lncRNA profiles of BC obtained from TCGA dataset, a risk model based on the pyroptosis-related lncRNAs for prognosis was constructed using univariate and multivariate Cox regression model, and least absolute shrinkage and selection operator. Patients were divided into high- and low-risk groups based on the risk model, and the prognosis value and immune response in different risk groups were analyzed. Furthermore, functional enrichment annotation, therapeutic signature, and tumor mutation burden were performed to evaluate the risk model we established. Moreover, the expression level and clinical significance of the selected pyroptosis-related lncRNAs were further validated in BC samples.

**Results:** 3,364 pyroptosis-related lncRNAs were identified using Pearson’s correlation analysis. The risk model we constructed comprised 10 pyroptosis-related lncRNAs, which was identified as an independent predictor of overall survival (OS) in BC. The nomogram we constructed based on the clinicopathologic features and risk model yielded favorable performance for prognosis prediction in BC. In terms of immune response and mutation status, patients in the low-risk group had a higher expression of immune checkpoint markers and exhibited higher fractions of activated immune cells, while the high-risk group had a highly percentage of TMB. Further analyses in our cohort BC samples found that RP11-459E5.1 was significantly upregulated, while RP11-1070N10.3 and RP11-817J15.3 were downregulated and significantly associated with worse OS.

**Conclusion:** The risk model based on the pyroptosis-related lncRNAs we established may be a promising tool for predicting the prognosis and personalized therapeutic response in BC patients.

## Introduction

Breast cancer (BC) is the most common malignant tumor and the leading cause of cancer-related death in women worldwide (Siegel et al., 2020). Owing to the development of local control and systematic treatment, including surgical resection, radiotherapy, systemic chemotherapy, in combination with targeted therapy, hormone replacement therapy, and other novel therapeutic methods, the survival outcome of BCs has significantly improved in the past decade (Harbeck and Gnant, 2017). Despite these efforts, the curative rate and prognosis of BC patients are still unsatisfactory, and up to 15% of cancer-related deaths occurred after treatment according to GLOBOCAN 2018 (Bray et al., 2018). In addition, the heterogeneity of BC results in the diversity of tumor evolution scenarios and traditional therapeutic response (Holm et al., 2017). Thus, there is an urgent requirement to identify novel sensitive biomarkers for predicting prognosis and developing targeted therapeutic agents in BC patients.

Pyroptosis is a novel programmed cell death mediated by the gasdermin family, accompanied by inflammatory and immune responses (Vande Walle and Lamkanfi, 2016). The relationship between pyroptosis and tumor remains mysterious, and the role of pyroptosis in cancer vary in different tissues and genetic backgrounds (Xia et al., 2019; Lu et al., 2021). On the one hand, pyroptosis as a type of cell death can inhibit the pathogenesis and development of tumor (Nagarajan et al., 2019; Tan et al., 2021); on the other hand, pyroptosis can form a suitable microenvironment for tumorigenesis and chemotherapeutic resistance by releasing multiple inflammatory mediators (Lee et al., 2019; Zhou and Fang, 2019; Li et al., 2021).

Recently, solid evidence suggested that pyroptosis plays a vital role in various tumors by regulating tumor cell proliferation, invasion, and migration. Lu et al. found that GSDME-mediated pyroptosis contributed to the drug response in a subset of lung cancer models, including KRAS-mutant, EGFR-altered, and ALK-rearranged adenocarcinomas (Lu et al., 2018). IL18, a pyroptosis-related inflammatory mediator, was observed to exert inflammation-dependent tumor-suppressive effects by promoting the differentiation, activity, and survival of tumor-infiltrating T cells in hepatocellular carcinoma (Markowitz et al., 2016). In terms of the innate immune microenvironment in tumor, Storr et al. found that macrophage-derived IL-1β promoted the migration of breast cancer cell and lymphatic endothelial cell adhesion, then contributed to the tumorigenesis and metastasis of BC (Storr et al., 2017; Tulotta et al., 2019). Moreover, IL-1β activated IRAK4 in cancer-associated fibroblasts, and then drove tumor fibrosis, chemoresistance, and poor prognosis in pancreatic cancer (Zhang et al., 2018). These results pinpoint pyroptosis as an unrecognized mechanism involved in the tumorigenesis and development in various tumors, which may have important implications for the clinical development and optimal application of anticancer therapeutics.

Long non-coding RNAs (lncRNAs), a cluster of RNAs that have no protein-encoding ability, have been widely accepted to play an important role in the pathogenesis and development of cancer (Fatica and Bozzoni, 2014; Bhan et al., 2017; Lin and Yang, 2018). Several studies proved the association between lncRNAs and the progression of breast cancer. Wang et al. found that H19 induced autophagy activation *via* the H19/SAHH/DNMT3B axis, which contributed to tamoxifen resistance in BC (Wang et al., 2019). Using lncRNA/mRNA microarray assays, Qin et al. observed that lnc030 maintained breast cancer stem cell stemness and tumorigenesis by stabilizing SQLE mRNA and increasing cholesterol synthesis (Qin et al., 2021). A study conducted by Xiu et al. showed that LINC02273 drove breast cancer metastasis by epigenetically upregulated AGR2 (Xiu et al., 2019). However, few efforts have been devoted to the role of pyroptosis regulators in the dysregulation of lncRNAs in breast cancer. Thus, by performing a comprehensive bioinformatics analysis, we aimed to construct and validate a risk model based on the pyroptosis-related lncRNAs to predict prognosis and immune response in breast cancer.

## Materials and Methods

### Data Acquisition and Preparation

The RNA sequence transcriptome profiling data and mutation data of BC patients with clinical features and survival information were downloaded from The Cancer Genome Atlas (TCGA) (https://portal.gdc.cancer.gov/repository). Then, using the Ensembl human genome browser (http://asia.ensembl.org/info/data/index.html) by the Perl program, the data were collated and annotated to protein-coding genes and lncRNAs. In total, 48,608 genes were annotated, in which 15,058 lncRNAs were identified.

### Selection of Pyroptosis Genes and Pyroptosis-Related lncRNAs

Based on previous review (Liu et al., 2021; Yu et al., 2021a), 23 genes (CASP1, CASP3, CASP4, CASP5, CASP6, CASP8, CASP9, CASP11, GSDMA, GSDMB, GSMDC, GSDMD, GSDME, NAIP, NLRC4, NLRP1, NLRP3, NLRP6, Pyrin, ASC, IL1β, IL18, AIM2) were defined as pyroptosis-related regulators. Pyroptosis-related lncRNAs were defined as lncRNAs that were significantly related to one or more of the 23 pyroptosis genes (|Pearson R| > 0.3 and *p* < 0.001) (Tang et al., 2021; Xu et al., 2021). Finally, 3,364 lncRNAs were identified as pyroptosis-related lncRNAs.

### Establishment and Verification of a Pyroptosis-Related lncRNA Signature

The entire TCGA set was randomized as a training set and a testing set with a ratio of 7:3. The training set (*N* = 764) was utilized to construct a risk model based on the pyroptosis-related lncRNAs, and the testing set (*N* = 327) was applied to validate this established model. Combined with BC survival information in TCGA, we screened the prognosis value of 3,364 pyroptosis-related lncRNAs in the training dataset by univariate Cox regression using the R package “survival”. Then using the R package “glmnet” (Tibshirani, 1997), we selected the most robust prognostic pyroptosis-related lncRNAs in LASSO Cox regression. Subsequently, multivariable Cox regression was applied to find the independent prognostic lncRNAs for overall survival (OS). Finally, a 10-pyroptosis-related lncRNAs risk signature was ultimately established (Yu et al., 2021b).

The risk score formula was calculated as follows:

$$\text{Risk}\;\text{score}=\Sigma \text{i}\;\;Coefficient\left(\mathrm{lncRNA}\right)\mathrm{\times Expression}\left(\mathrm{lncRNA}\right){\;}^{[29]}$$

where coefficient (lncRNA) was the coefficient of lncRNAs correlated with survival and expression (lncRNA) was the expression of lncRNAs. According to the median risk score, patients were divided into low- and high-risk groups.

### Evaluation of the Immune Landscape

The abundance of 22 immunocytes between the low-risk and high-risk groups were calculated through CIBERSORT algorithm (Newman et al., 2015). The immune-related efficiency was estimated using the “MCPcounter” package (Becht et al., 2016). Immune and stromal scores of BC patients were estimated applying the “estimate” package (Yoshihara et al., 2013). Besides, the expression of key immune profiles between the low-risk and high-risk groups was compared using the Wilcoxon test.

### Exploration of the Overall Gene Mutation and Tumor Mutation Burden in Different Risk Groups

Using the R package maftools (Mayakonda et al., 2018), the overall gene mutation status was analyzed and summarized in the high- and low-risk groups. Then, TMB scores based on the TGCA somatic mutation data were calculated to evaluate the mutation status between different risk groups.

### Exploration of Potential Compounds Targeting the Pyroptosis-Related lncRNA Model

To identify potential drugs targeting the lncRNA-based risk model for treating BC patients, we estimated the therapeutic response based on the half-maximal inhibitory concentration (IC_50_) of various molecular data available in the CellMiner database for each sample (Reinhold et al., 2012).

### Functional Enrichment Analysis

Gene set enrichment analysis (GSEA) was performed to investigate the potential biological process and cellular pathway between the low- and high-risk groups through the Clusterprofile package (Subramanian et al., 2005). The FDR *q* <0.25 and *p* <0.05 were considered statistically significant.

### Independence of the Pyroptosis-Related lncRNA Model

The Kaplan–Meier survival curve was performed to compare the survival diversities of the high-risk and low-risk groups. Univariable and multivariable Cox regression analyses were conducted to test whether the risk model we constructed was an independent risk factor for survival in BC patients.

### Establishment and Validation of a Prognostic Nomogram

To predict the prognosis of BC patients, a nomogram based on risk model and other clinicopathologic features were constructed to predict the 1-, 3-, and 5-year OS using the R package “rms” (Zhang and Kattan, 2017). The concordance index (C-index) and calibration plots were applied to reflect the predictive accuracy of the prognostic nomogram we constructed. The area under the time-dependent receiver operating characteristic curve (AUC) were performed to evaluate the sensitivity and specificity of the prognostic nomogram in both the training and validation sets.

### Validation of the Bioinformatics Results Using RT-qPCR Assay

A total of 133 paired BC tissues (T) and adjacent normal tissues (N) were obtained from Sir Run Run Shaw Hospital of Zhejiang University from 2014 to 2017. Total RNA was extracted using TRIzol reagent (Invitrogen, USA). Reverse transcription was conducted using PrimeScript RT MasterMix (Takara, China). qRT-PCR was performed using SYBR Green PCR MasterMix (Takara, China). The qRT-PCR primers are listed in Supplementary Table S1.1; target lncRNA expression was normalized to those of GAPDH. The workflow of this study is shown in Supplementary Figure S1.

## Results

### Identification of Pyroptosis-Related lncRNAs

The matrix expression of 23 pyroptosis genes and 15,058 lncRNAs were abstracted from the TCGA database. Then, applying Pearson’s correlation analysis with a criteria of |Pearson R| >0.3 and *p* < 0.001, 3,364 lncRNAs were identified closely related to the 23 pyroptosis-related regulators, and these lncRNAs were defined as pyroptosis-related lncRNAs.

### Construction and Validation of a Risk Model Based on the Pyroptosis-Related lncRNAs

**FIGURE 1:**
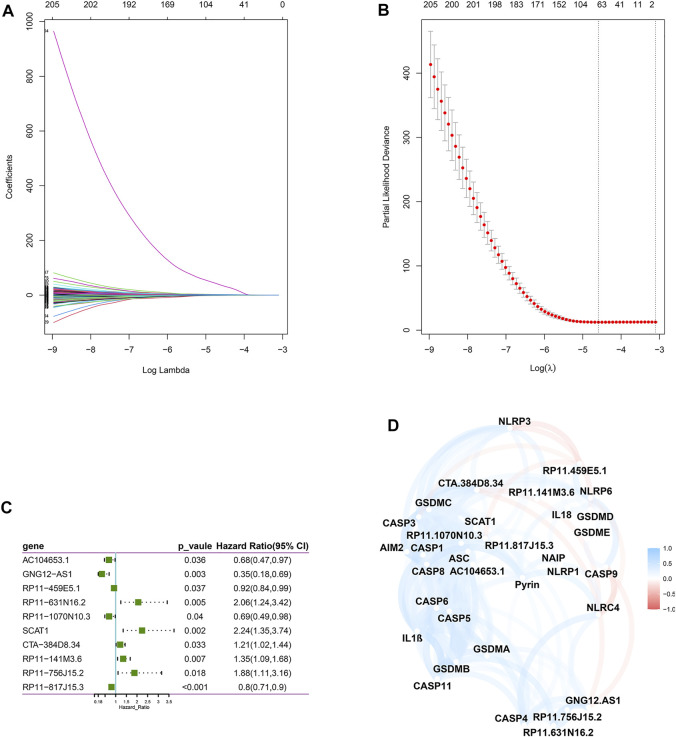
Construction of a risk model for BC patients based on the pyroptosis-related lncRNAs. **(A,B)** The LASSO coefficient profile was constructed from 64 prognostic pyroptosis-related lncRNAs based on the minimum criteria for OS with 10-fold cross-validation. **(C)** Multivariate Cox regression analysis showed 10 independent prognostic pyroptosis-related lncRNAs. **(D)** Correlation network between the 23 pyroptosis genes and the 10 prognostic pyroptosis-related lncRNAs.

**FIGURE 2:**
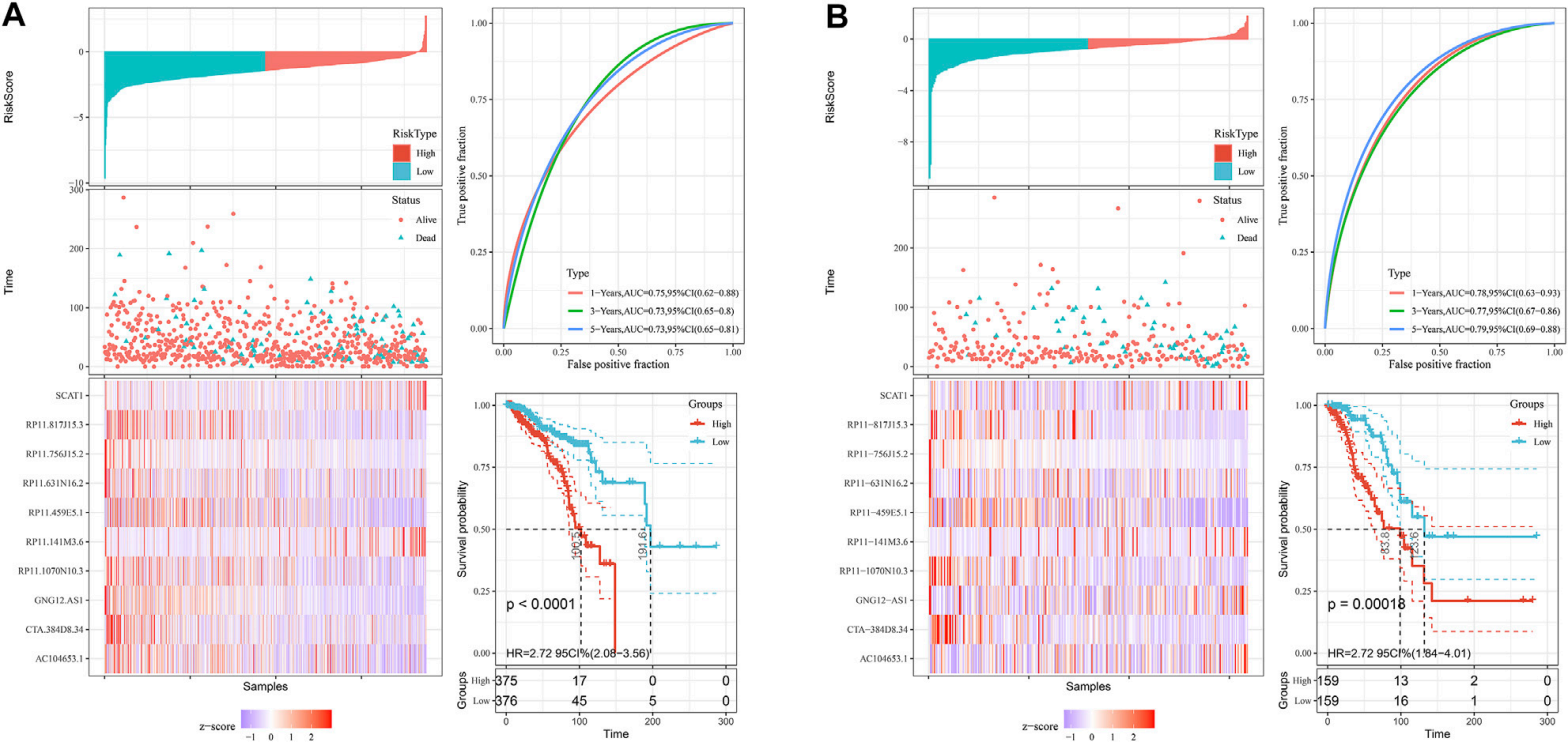
The distribution of risk score, survival analysis, and the expression level of the 10 pyroptosis-related lncRNAs in the training **(A)** and testing sets **(B)**.

Next, we screened prognostic-related lncRNAs from 3,364 pyroptosis-related lncRNAs in the training set using univariate Cox regression analysis. In total, 381 pyroptosis-related lncRNAs were significantly correlated with OS. Sixty-four pyroptosis-related lncRNAs were selected by performing the LASSO Cox analysis (Figures 1A,B). Next, 10 pyroptosis-related lncRNAs were found as independent predictors for OS in the training set using multivariate Cox ratio hazard regression analysis (Figure 1C). Then, a risk model was constructed and patients were clustered into low- and high-risk groups based on their risk scores (Supplementary Table S1.2). In addition, the correlation between the pyroptosis-related lncRNAs and pyroptosis genes was analyzed, which is shown in Figure 1D. The distribution of risk score, survival analysis, and the expression level of the 10 pyroptosis-related lncRNAs in the training and testing sets are shown in Figures 2A,B.

### Estimation of the Tumor Immune Microenvironment

**FIGURE 3:**
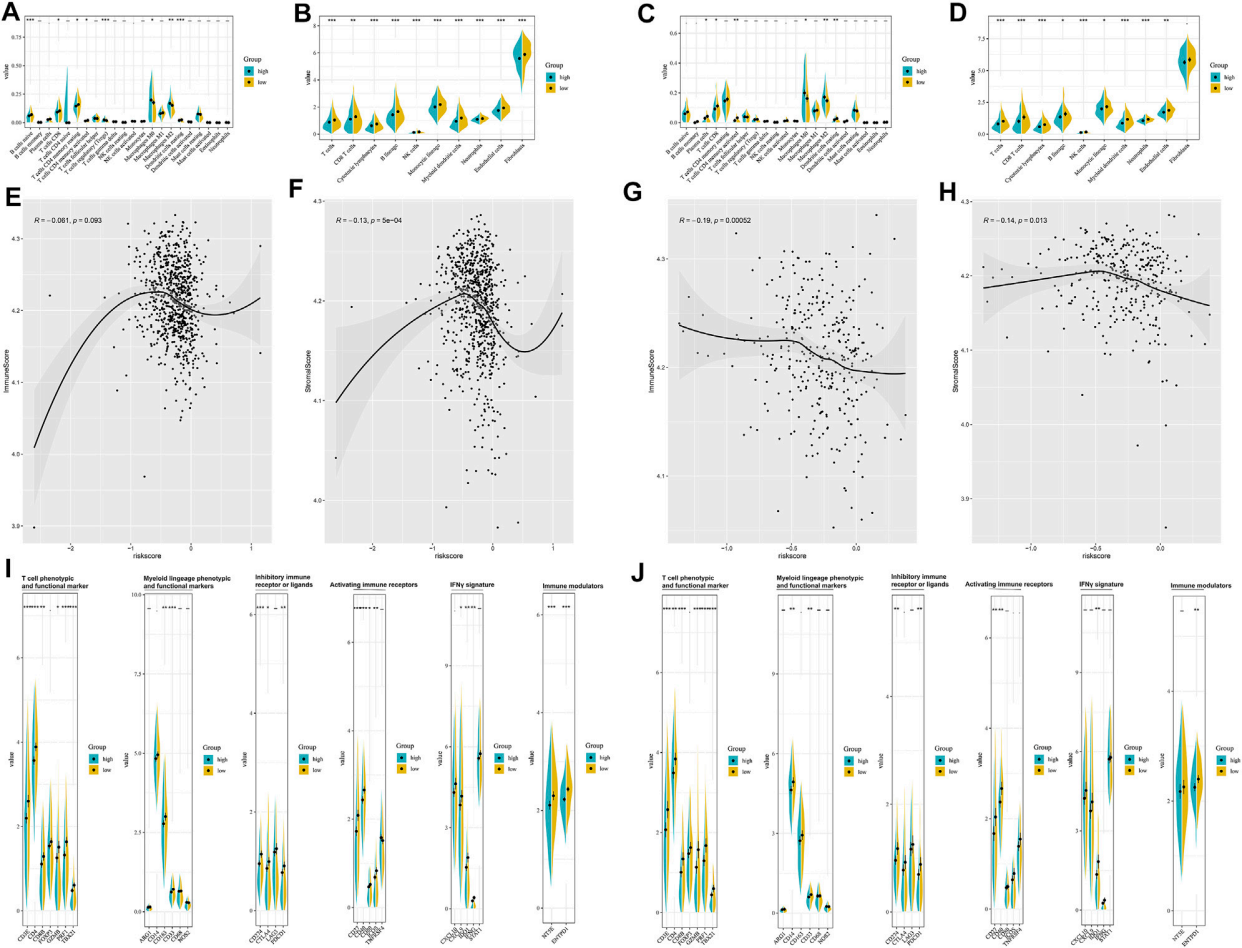
Estimation of tumor immune microenvironment in the pyroptosis-related lncRNA-based risk groups. CIBERSORT analyses between different risk groups in the training set **(A)** and testing set **(B)**. MCP-counter analyses between different risk groups in the training set **(C)** and testing set **(D)**. ESTIMATE analyses between different risk groups in the training set **(E,F)** and testing set **(G,H)**. The expression of immune profiles between different risk groups in the training set **(I)** and testing set **(J)**.

The enrichment level and activity of infiltrating immune cells between the different risk groups were further analyzed. CIBERSORT algorithm (Figures 3A,B) confirmed that patients in the low-risk group were qualified with more antitumoral immune cells (CD8^+^ T cells, activated memory CD4^+^ T cells), while patients in the high-risk group were characteristics of more regulatory T cells and M2 macrophages. MCP-counter (Figures 3C,D) suggested that patients in the low-risk group had a higher level of activated immune cells (CD8^+^ T cells, cytotoxic lymphocytes, B cells, and NK cells). ESTIMATE algorithm indicated that the risk model was negatively correlated with the immune score and the stromal score (Figures 3E–H). Besides, the low-risk and high-risk groups showed prominent differences in the expression of immune profiles (Figures 3I,J), and a higher expression of immune-related profiles, like T cell phenotypic and functional marker (CD3E, CD4, CD8B, FOXP3, GZMB, PRF1, and TBX21), activating immune receptors (CD27, CD40, CD80, ICOS, and TNFRSF4), IFNγ signature (CXCL9, CXCL10, IDO1, IFNG, and STAT1), and the immune checkpoint markers (CTLA4, CD274, PDCD1), was observed in the low-risk group, which indicated that the pyroptosis-based lncRNA signature might serve as an effective indicator for immunotherapeutic response.

### Overall Gene Mutation and Tumor Mutation Burden Analysis

**FIGURE 4:**
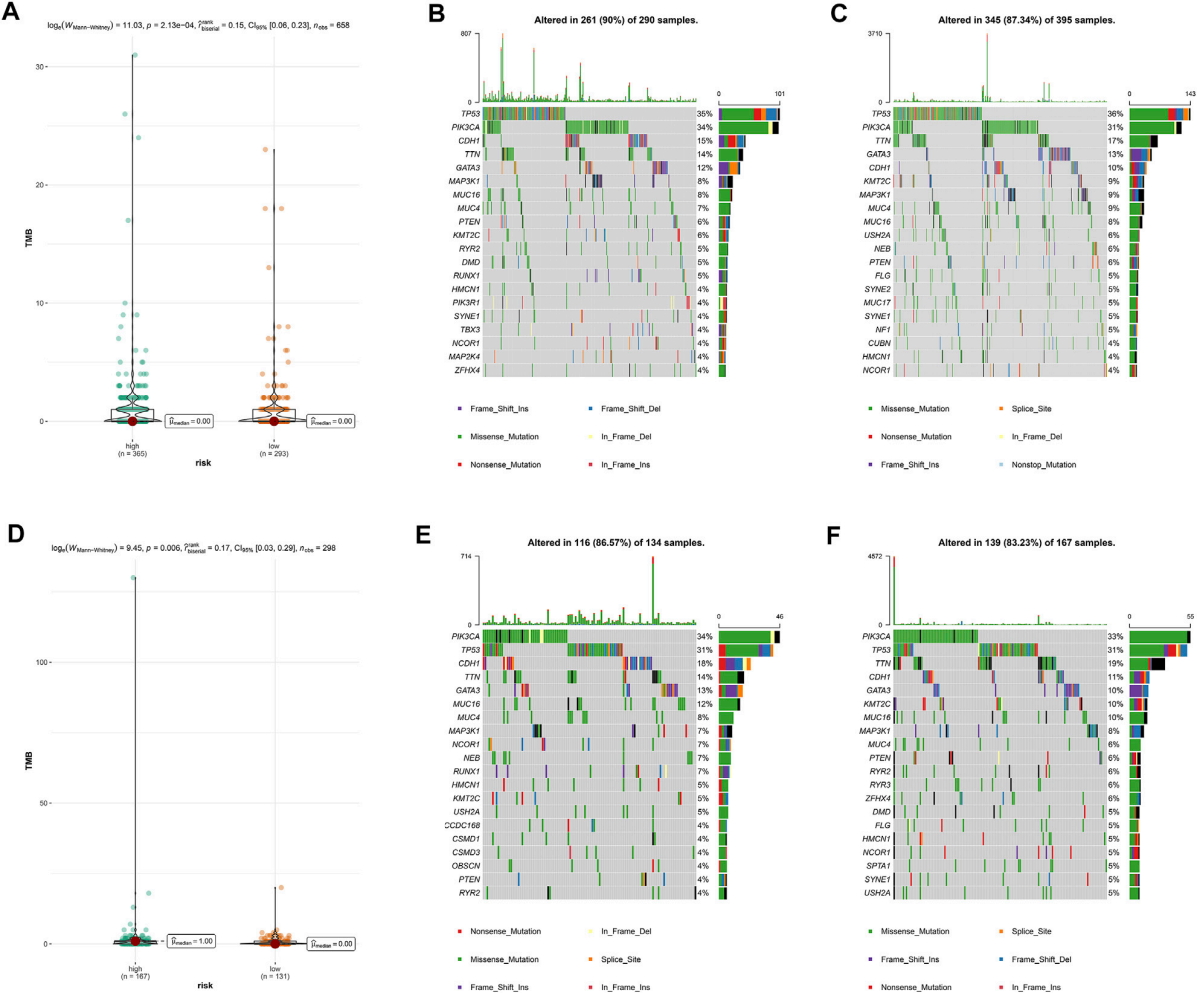
Overall gene mutation and tumor mutation burden analysis based on the pyroptosis-related lncRNAs model. The difference of tumor mutational burden between the high- and low-risk subtypes in the training set **(A)** and testing set **(D)**. Waterfall plots of top 20 mutated genes in the low-/high-risk groups in the training set **(B,C)** and testing set **(E,F)**.

Then, we calculated TMB scores according to tumor-specific mutated genes. Patients in the high-risk group showed a significantly higher TMB than their counterpart in the low-risk group (Figures 4A,D). Using the R package maftools, we analyzed the overall gene mutation in different risk groups. The top 20 driver genes with the highest alteration frequency were depicted by waterfall plots in the low-/high-risk groups in the training set (Figures 4B,C) and testing set (Figures 4E,F).

### Identification of Novel Candidate Compounds Targeting the Pyroptosis-Related lncRNA Model

**FIGURE 5:**
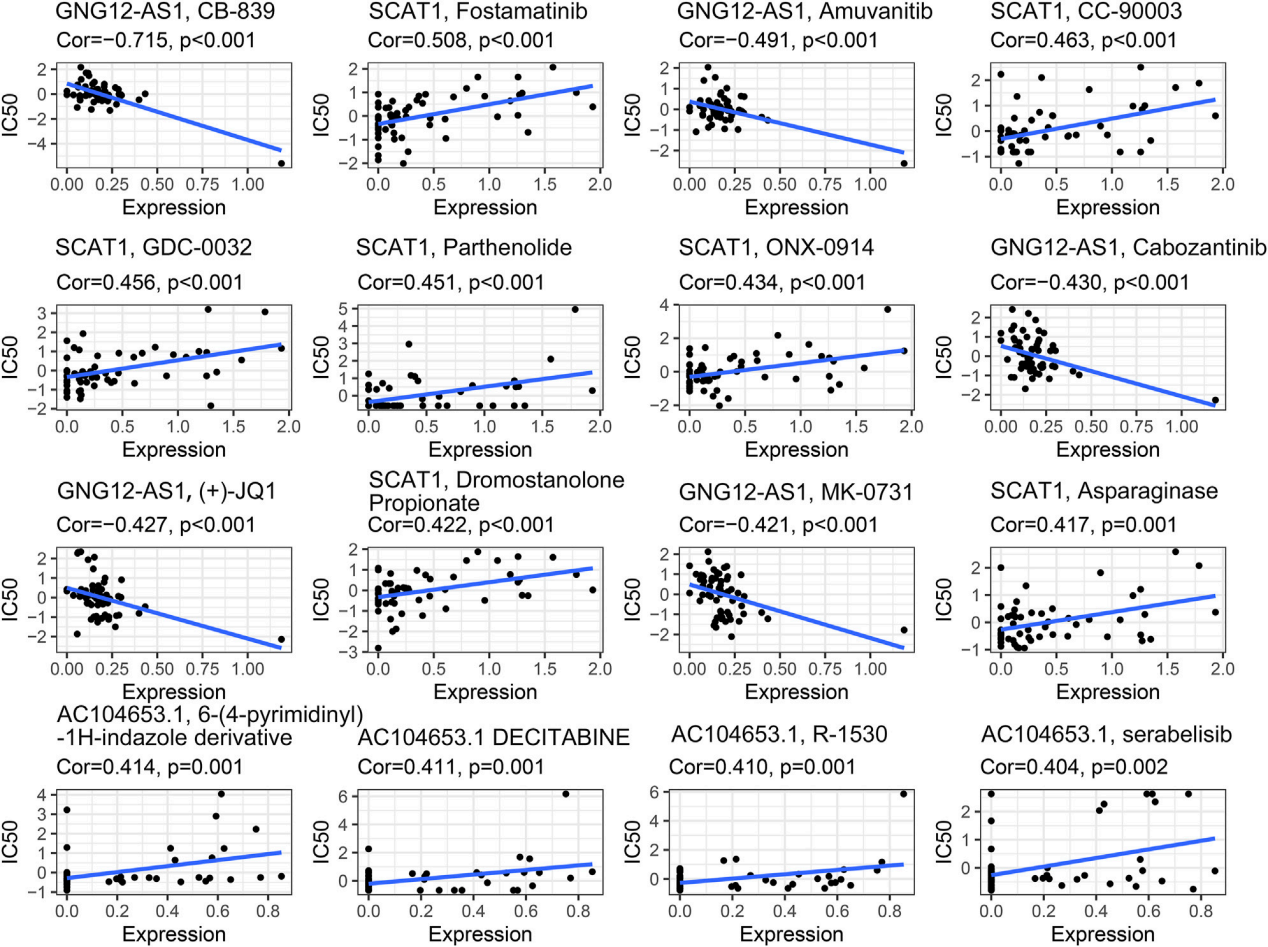
Identification of novel candidate compounds targeting the pyroptosis-related lncRNA model.

To identify potential compounds for the treatment of BC, we calculated the IC_50_ of compounds obtained from the CellMiner database. Robust negative correlation has been found between the expression level of GNG12-AS1 with IC_50_ of CB-839, amuvanitib, cabozantinib, (+)-JQ1, and MK-0731 (all *p* < 0.001). A significant positive correlation was observed between the expression of SCAT1 and IC_50_ of fostamatinib, CC-90003, GDC-0032, parthenolide, ONX-0914, asparaginase, and dromostanolone propionate (all *p* < 0.005). The expression level of AC104653.1 was significantly positively associated with the IC_50_ of 6-(4-pyrimidinyl)-1H-indazole derivative, decitabine, R-1530, and serabelisib (all *p* < 0.005), which is shown in Figure 5.

### Functional Enrichment Analysis

**FIGURE 6:**
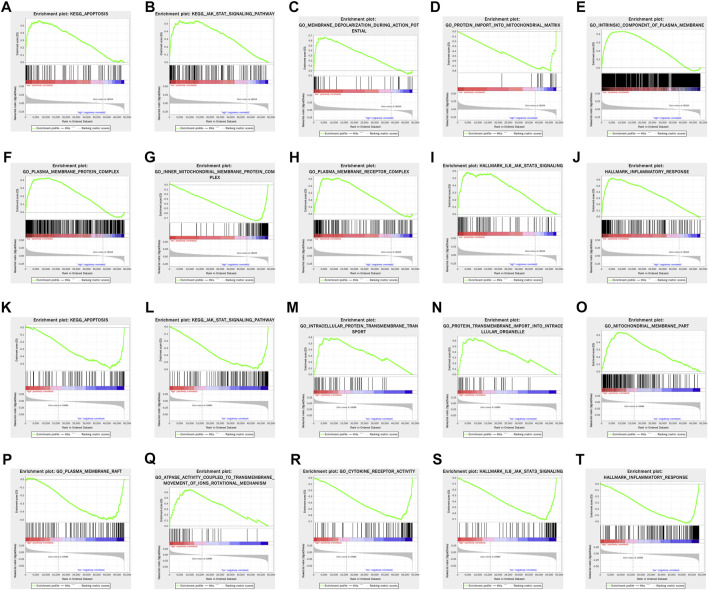
GSEA of enriched potential biological process and cellular pathway between the high- and low-risk groups in the training set **(A–J)** and testing set **(K–T)**.

Gene set enrichment analysis (GSEA) revealed that apoptosis and JAK-STAT signaling pathway were significantly enriched in the high-risk group in both training and testing sets. In terms of gene annotation (GO) analysis, protein transmembrane import into intracellular organelle, mitochondrial membrane part, and plasma membrane receptor complex were the most relevant biological process (BP), cellular component (CC), and molecular function (MF) of pyroptosis-related lncRNAs, respectively. Pertaining to cancer hallmark, IL6-JAK-STAT3 signaling pathway and inflammatory response were the most relevant cancer hallmarks (Figure 6).

### Evaluation of the Prognostic Value of the Risk Model

To further evaluate the prognostic value of the risk model, univariate and multivariate Cox regression models that contained the clinicopathologic features and the risk model were analyzed in the training and testing sets. As shown in Supplementary Table S2, the risk model was an independent risk factor for OS in BC patients. Moreover, the expression level and prognostic value of these pyroptosis-related lncRNAs were further analyzed using GEPIA dataset (http://gepia.cancer-pku.cn/). Kaplan–Meier survival analyses showed the survival diversity of the pyroptosis-related lncRNAs (Supplementary Figure S2). The expression of these pyroptosis-related lncRNA signatures is depicted in Supplementary Figure S2. Results showed that RP11-756J15.2, RP11-1070N10.3, RP11-817J15.3, RP11-459E5.1, and RP11-141M3.6 were lowly expressed in tumor tissues and positively associated with OS in BC patients.

### Construction and Evaluation of the Pyroptosis-Related lncRNA-Based Nomogram

**FIGURE 7:**
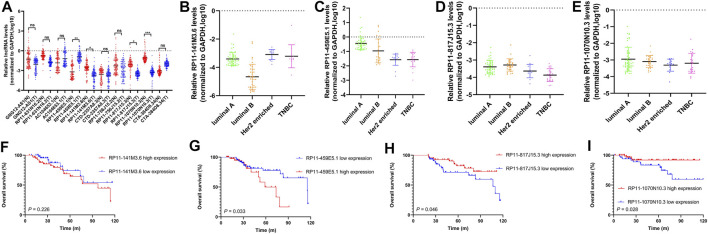
The expression levels and prognostic value of 10 selected pyroptosis-related lncRNAs in our cohort. **(A)** Comparison of expression levels of 10 selected pyroptosis-related lncRNAs in tumor tissues (T) and adjacent normal tissues (N) by RT-qPCR assay. The expression level of RP11-141M3.6, RP11-459E5.1, RP11-817J15.3, and RP11-1070N10.3 in different breast cancer subtypes, respectively **(B–E)**. Kaplan–Meier curve shows the survival diversity between different expressions of RP11-141M3.6 **(F)**, RP11-459E5.1 **(G)**, RP11-817J15.3 **(H)**, and RP11-1070N10.3 **(I)** in our cohort. Non-significant (ns) *p* > 0.05, *p < 0.05, **p < 0.01, and ***p < 0.001.

A nomogram comprising the risk model and clinicopathologic features was established to predict the 1-, 3-, and 5-year OS. By comparison with other clinicopathologic parameters, the risk model showed predominant predictive ability in the nomogram (Figure 7A). The AUC value (Figures 7B,C) and calibration plots (Figures 7D–I) showed excellent consistency between the actual and nomogram-predicted survival probabilities for 1-, 3-, and 5-year OS in the training and testing sets.

### The Expression Levels and Prognostic Value of 10 Selected Pyroptosis-Related lncRNAs in Our Cohort

**FIGURE 8:**
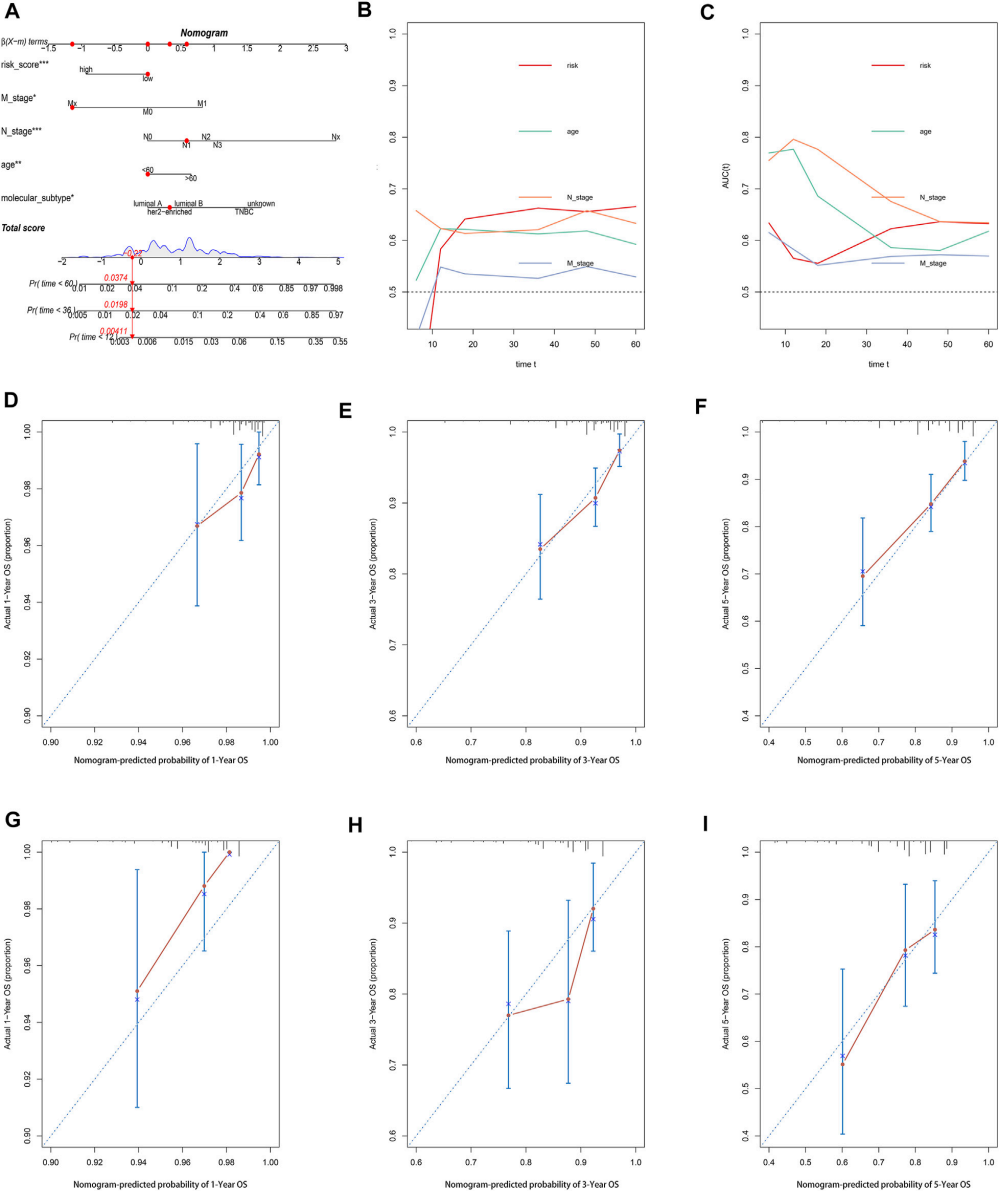
Construction and evaluation of a pyroptosis-related lncRNA-based nomogram. **(A)** The nomogram predicts the probability of 1-, 3-, and 5-year OS for individual patients. AUC values of the risk score and clinical characteristics in the training set **(B)** and testing set **(C)**. Calibration plots evaluate the prediction accuracy of the nomogram in the training set **(D–F)** and testing set **(G–I)**.

The expression levels of 10 selected pyroptosis-related lncRNAs (AC104653.1, GNG12-AS1, RP11-141M3.6, RP11-631N16.2, RP11-459E5.1, RP11-756J15.2, RP11-817J15.3, RP11-1070N10.3, CTA-384D8.34, and CTD-2357A8.3) were examined by qRT-PCR. In detail, RP11-141M3.6, RP11-1070N10.3, and RP11-817J15.3 were downregulated, while RP11-459E5.1 was significantly upregulated in tumor tissues compared with that in the paired normal tissues (Figure 8A). The four differentially expressed lncRNAs were further analyzed in different breast cancer subtypes (Figures 8B–E). Importantly, Kaplan–Meier survival analysis showed that high expression of RP11-459E5.1 was significantly associated with worse OS (Figure 8G), while RP11-1070N10.3 and RP11-817J15.3 high expression (Figures 8H,I) were correlated with better survival in breast cancer, which is in accord with the bioinformatics results.

## Discussion

Breast cancer is the most frequent malignant tumor and the leading cause of cancer-related death in women globally. Although dramatic improvements in early diagnosis and effective treatment have been made for this malignant tumor, a considerable proportion of patients still succumb to metastasis and recurrence due to therapeutic failure. It is well established that breast cancer displays extreme heterogeneity in histology and molecular analysis, which contributes to significant diversities in incidence, malignant procession, treatment response, and prognosis (Holm et al., 2017; Yeo and Guan, 2017). Thus, more insights are required to identify critical signaling molecules that contribute to the tumorigenesis and malignant procedure of breast cancer.

Pyroptosis is a novel type of programmed cell death characterized by gasdermin-mediated and proinflammatory factor release (Shi et al., 2015). Increasing evidence suggested that pyroptosis may play a critical role in the pathogenesis and development of various tumors, including BC. However, studies on the pathological role of pyroptosis in BC progression remain limited. Similarly, lncRNAs, the largest class of noncoding RNA, modulate chromatin functions through interactions with DNA, RNA, and proteins (Qian et al., 2019; Statello et al., 2021). Numerous studies have explored the correlation of lncRNA with various cancers, including BC. However, studies on the biological mechanisms and prognostic biomarkers of BC concerning pyroptosis-related lncRNAs are still lacking.

In the present study, we were inspired by the biological function of pyroptosis and lncRNAs in BC; thus, we attempted to construct a risk model based on pyroptosis-related lncRNAs for predicting prognosis and immune response in BC patients. First, we identified 3,364 pyroptosis-related lncRNAs in the TCGA cohort. Then, using univariate and multivariate Cox regression analyses, and LASSO cox regression analysis, a risk model based on 10 pyroptosis-related lncRNAs were constructed and patients were classified into high- and low-risk groups based on the median risk score. Results showed that the high-risk group had apparently poorer OS than the low-risk group. Multivariate Cox regression analysis showed that the pyroptosis-related lncRNA model was an independent risk factor of OS. Then, a nomogram was established to predict the 1-year, 3-year, and 5-year OS of BC patients. The calibration plots and AUC value showed excellent consistency between the actual and nomogram-predicted survival probabilities for 1-, 3-, and 5-year OS in the training and testing sets. Moreover, the expression level and clinical significance of the selected pyroptosis-related lncRNAs were further validated in our cohort, which is in accord with the bioinformatics results.

Next, we explored the relationship of the risk model and tumor microenvironment in BC. Results showed that patients in the low-risk group had a higher expression of immune-checkpoint markers (like CTLA4, CD274, and PDCD1) as compared with the high-risk group. Similarly, we observed that the low-risk group had higher infiltration levels of activated immune cells than their counterpart (CD8^+^ T cells, B cells, NK cells, etc.). The results were consistent with previous findings that immune checkpoints executed a vital role on tumorigenesis and development in tumors by inducing tumor immune-suppressive activities, and patients with high PD-L1 expression in tumor cells and stromal immune cells are more likely to respond to chemotherapy and immunotherapy (Gibson, 2015; Kwapisz, 2021). Liu et al. reported that the aberrant expression of CTLA4 and PDCD1 was associated with tumorigenesis and immunocyte infiltration in pan-cancer, including breast cancer (Liu et al., 2020). Another study conducted by Park et al. showed that the expression level of CD274 was associated with prognosis in breast cancer patients who received neoadjuvant chemotherapy (Park et al., 2020). Moreover, Tekpli et al. (2019) found an immune infiltration–based subtype of breast cancer to predict therapeutic response and prognosis in breast cancer patients.

Furthermore, we estimated the TMB and overall gene mutations in different risk groups. TMB is defined as the total number of somatic mutations per megabase of interrogated genomic sequence, which related to the emergence of neoantigens that trigger antitumor immunity (Sha et al., 2020). Recent studies revealed that breast cancer patients with high TMB were more likely to benefit from PD-L1 inhibitors (Thomas et al., 2018; Barroso-Sousa et al., 2020; Chumsri et al., 2020). The results of our study showed that the difference in the amount of overall gene mutations between the high- and low-risk groups, and the TMB in the low-risk group exceeded that in high-risk group. In terms of chemotherapy response, the expression of GNG12-AS1 was negatively associated with IC_50_ of CB-839, amuvanitib, cabozantinib, (+)-JQ1, and MK-0731. Also, significant positive correlations were observed between the expression of SCAT1(CTD-2357A8.3) and AC104653.1 with IC_50_ of other agents, which may be potential compounds for the treatment of BC by targeting these specific pyroptosis-related lncRNAs.

In addition, as revealed in the GSEA results, the tumor functional patterns including apoptosis and JAK-STAT signaling pathways were enriched in the high-risk group. Pertaining to cancer hallmark, IL6-JAK-STAT3 signaling pathway and inflammatory response were the most relevant cancer hallmark. It is well established that JAK-STAT signaling is involved in breast cancer cell proliferation, metastasis, and chemotherapeutic sensitiveness. For instance, Wang et al. reported that CircNOL10 suppressed breast cancer progression by sponging miR-767-5p to regulate SOCS2/JAK/STAT signaling (Wang et al., 2021). Several studies found that JAK-STAT signaling was involved in the chemotherapeutic and endocrine therapeutic resistance in breast cancer (Lui et al., 2017; Zhu et al., 2020). Considering that, the 10 pyroptosis-related lncRNAs and their relative pathways may be involved in the tumorigenesis and development of breast cancer.

Among the lncRNA signatures, GNG12-AS1 has been found to coregulate with DIRAS3, and then inhibit cell cycle progression and migration in various tissues (Stojic et al., 2016). Moreover, GNG12-AS1 caused allele-specific splicing in breast cancer, which may contribute to the tumorigenesis and development of breast cancer (Niemczyk et al., 2013). Xiang et al. observed that GNG12-AS1 induced glioma cell proliferation and migration through AKT/GSK-3β/β-catenin signaling (Xiang et al., 2020). Zheng et al. found that SCAT1(CTD-2357A8.3) served as a predictive biomarker for pathologic complete response of chemotherapy in esophageal squamous cell carcinoma (Zhang et al., 2020). A previous study by Fan et al. found that SCAT1(CTD-2357A8.3) could be a novel prognostic biomarker for esophageal cancer (Fan and Liu, 2016). Similarly, Lei et al. established a risk model, which contained AC104653.1 to predict the prognosis of glioblastoma, and results showed that the model was a powerful tool for survival prediction in this malignant tumor (Lei et al., 2018). Furthermore, pan-cancer analysis of S-phase enriched lncRNAs identified that SCAT1(CTD-2357A8.3) was differentially expressed in several cancers, SCAT1(CTD-2357A8.3) induced cell proliferation and correlated with poor prognosis in lung cancer (Ali et al., 2018), and these publications provided a novel biological function and mechanism of these lncRNAs in tumors.

It is an undeniable fact that several limitations existed in this study. Due to lack of available data about lncRNAs in other databases, like the Gene Expression Omnibus (GEO) database, we could not validate the results of our study in other public datasets. In this background, we collected BC samples in our cohort to further explore the expression level and clinical significance of these pyroptosis-related lncRNAs, which validated the clinical significance of the selected pyroptosis-related lncRNAs. However, further experimental studies are needed to elucidate the underlying biological function and mechanism of these pyroptosis-related lncRNAs in breast cancer.

In conclusion, the established pyroptosis-related lncRNA model provides a new method for prognostic prediction in BC patients, and may help elucidate the important role of these pyroptosis-related lncRNAs in the tumorigenesis and development of breast cancer. In addition, our study provides new insight in identifying BC patients who may benefit from immunotherapy.

## Data Availability

The original contributions presented in the study are included in the article/Supplementary Material, further inquiries can be directed to the corresponding author.

## Supplementary Material

Supplementary Figure S1 Kaplan–Meier curves to depict the survival diversity of the pyroptosis-related lncRNAs.

Supplementary Figure S2 The expression of pyroptosis-related lncRNAs signature in the TCGA dataset.
