# Supplementary material for: Pyroptosis-Related lncRNAs Predict the Prognosis and Immune Response in Patients With Breast Cancer
Source: Front Genet. 2022 Mar 14;12:792106. doi: 10.3389/fgene.2021.792106 (PMC8963933; doi:10.3389/fgene.2021.792106)
Supplement: Supplementary file 1 [file Table1.DOCX]

Table S1.1 the primers of selected pyroptosis-related lncRNAs.

| lncRNA |  | Sequence (5' -> 3') |
| --- | --- | --- |
| AC104653.1 | Forward Primer | CTATAGACAGTGCCAGTGTG |
|  | Reverse Primer | GCTGTAATTGCTTTCCCAG |
| GNG12-AS1 | Forward Primer | AGTGGACGCTTATCTTCCC |
|  | Reverse Primer | CTAGGGCTTGATGTCCTCC |
| RP11-459E5.1 | Forward Primer | CAAACAGGCCAGTTCACAC |
|  | Reverse Primer | AAATGGCTGTAGTCCCAGG |
| RP11-631N16.2 | Forward Primer | TCTCTAAGGTTCAAGAGATTCTCC |
|  | Reverse Primer | TCAGGAGTTTGAGACCAGC |
| RP11-1070N10.3 | Forward Primer | CAGCAGAAACATCCTCCCA |
|  | Reverse Primer | AAGGTTAAAGGGTTGGCGT |
| CTD-2357A8.3 | Forward Primer | GCTTTAACAACAGAAAGACCC |
|  | Reverse Primer | GCAGATGTCTTTCATTAAGCTG |
| CTA-384D8.34 | Forward Primer | ATCCACGTCATTGAGAAACG |
|  | Reverse Primer | AGCACTTCCATGTCATCTG |
| RP11-141M3.6 | Forward Primer | CTATTGAACACGGCAGAGC |
|  | Reverse Primer | ATGAGCTACCTCAACTTTGC |
| RP11-756J15.2 | Forward Primer | AAAGCAGAAGAAGGCAATACC |
|  | Reverse Primer | GACTGTGAGACATTCCTAAGAG |
| RP11-817J15.3 | Forward Primer | GGAGAATCAGGTTCTTATAGGG |
|  | Reverse Primer | TTCCACTTCAGTCATTGCA |

Table S1.2 the factors in the risk model.

| RP11.817J15.3 | -0.190259208 |
| --- | --- |
| AC104653.1 | -0.099651498 |
| GNG12.AS1 | -1.179597326 |
| RP11.459E5.1 | -0.024170257 |
| SCAT1(CTD-2357A8.3) | 0.581714107 |
| RP11.631N16.2 | 0.458076688 |
| RP11.1070N10.3 | -0.07066104 |
| CTA.384D8.34 | -0.124062433 |
| RP11.141M3.6 | 0.094858583 |
| RP11.756J15.2 | -0.162610353 |
