# Supplementary material for: Pyroptosis-Related lncRNAs Predict the Prognosis and Immune Response in Patients With Breast Cancer
Source: Front Genet. 2022 Mar 14;12:792106. doi: 10.3389/fgene.2021.792106 (PMC8963933; doi:10.3389/fgene.2021.792106)
Supplement: Supplementary file 3 [file Table2.DOCX]

| **Table S2.1 Univariable and multivariable Cox regression analyses for OS in the training set.** | | | | |  |
| --- | --- | --- | --- | --- | --- |
| \| Variables \|  \| Univariate analysis \| \| Multivariate analysis \| \| \| --- \| --- \| --- \| --- \| --- \| --- \| \|  \| HR (95% CI) \| *P*-value \| HR (95% CI) \| *P*-value \| \| Age at diagnosis (years) \| <60 \| 1 (reference) \| 1 \| 1 (reference) \| 1 \| \|  \| ≥60 \| 1.87 (1.23-2.82) \| 0.003 \| 1.89 (1.22-2.94) \| 0.005 \| \|  \| Unknown \| NA \| NA \| NA \| NA \| \| Histologic type \| IDC \| 1 (reference) \| 1 \|  \|  \| \|  \| ILC \| 0.80 (0.45-1.41) \| 0.432 \|  \|  \| \|  \| Other \| 1.17 (0.47-2.93) \| 0.73 \|  \|  \| \| T stage \| T1 \| 1 (reference) \| 1 \| 1 (reference) \| 1 \| \|  \| T2 \| 1.09 (0.66-1.80) \| 0.744 \| 0.94 (0.56-1.57) \| 0.8 \| \|  \| T3 \| 1.65 (0.86-3.17) \| 0.133 \| 1.09 (0.53-2.26) \| 0.818 \| \|  \| T4 \| 3.01 (1.27-7.14) \| 0.013 \| 1.31 (0.50-3.41) \| 0.587 \| \| N stage \| N0 \| 1 (reference) \| 1 \| 1 (reference) \| 1 \| \|  \| N1 \| 1.68 (1.02-2.76) \| 0.043 \| 1.74 (1.04-2.92) \| 0.035 \| \|  \| N2 \| 2.52 (1.31-4.85) \| 0.006 \| 2.48 (1.24-4.95) \| 0.01 \| \|  \| N3 \| 5.52 (2.98-10.22) \| <0.001 \| 4.01 (1.88-8.56) \| <0.001 \| \| M satge \| M0 \| 1 (reference) \| 1 \| 1 (reference) \| 1 \| \|  \| M1 \| 5.22 (2.86-9.52) \| <0.001 \| 2.55 (1.26-5.14) \| 0.009 \| \|  \| Unknown \| 0.60 (0.24-1.50) \| 0.275 \| 0.50 (0.20-1.26) \| 0.139 \| \| Risk score \| Low \| 1 (reference) \| 1 \| 1 (reference) \| 1 \| \|  \| High \| 0.58 (0.38-0.89) \| 0.012 \| 0.56 (0.36-0.88) \| <0.001 \| \| ER \| Negative \| 1 (reference) \| 1 \|  \|  \| \|  \| Positive \| 0.65 (0.42-1.04) \| 0.072 \|  \|  \| \|  \| Unknown \| 1.93 (0.79-4.73) \| 0.149 \|  \|  \| \| PR \| Negative \| 1 (reference) \| 1 \|  \|  \| \|  \| Positive \| 0.78 (0.51-1.21) \| 0.266 \|  \|  \| \|  \| Unknown \| 2.26 (0.94-5.42) \| 0.069 \|  \|  \| \| HER2 \| Negative \| 1 (reference) \| 1 \|  \|  \| \|  \| Positive \| 1.02(0.53-1.96) \| 0.949 \|  \|  \| \|  \| Unknown \| 1.74 (0.74-2.89) \| 0.053 \|  \|  \| \| Molecular subtype \| Luminal A \| 1 (reference) \| 1 \| 1 (reference) \| 1 \| \|  \| Luminal B \| 0.54 (0.27-1.25) \| 0.095 \| 0.88 (0.64-1.66) \| 0.083 \| \|  \| Her2 enriched \| 1.56 (0.64-3.79) \| 0.312 \| 1.60 (0.61-4.19) \| 0.338 \| \|  \| TNBC \| 2.08 (1.22-3.47) \| 0.001 \| 2.38 (1.46-4.12) \| 0.001 \| \|  \| Unknown \| 0.77 (0.39-1.89) \| 0.14 \| 0.70 (0.45-1.30) \| 0.124 \| |  |  |  |  |  |

**Table S2.2** **Univariable and multivariable Cox regression analyses for OS in the testing set.**

| Variables |  | Univariate analysis | | Multivariate analysis | |
| --- | --- | --- | --- | --- | --- |
|  |  | HR (95% CI) | *P*-value | HR (95% CI) | *P*-value |
| Age at diagnosis (years) | <60 | 1 (reference) | 1 | 1 (reference) | 1 |
|  | ≥60 | 2.03 （1.21-3.42） | 0.008 | 2.07 (1.22-3.52) | 0.007 |
|  | Unknown | NA | NA | NA | NA |
| Histologic type | IDC | 1 (reference) | 1 |  |  |
|  | ILC | 0.80 (0.45-1.41) | 0.432 |  |  |
|  | Other | 1.17 (0.47-2.93) | 0.73 |  |  |
| T stage | T1 | 1 (reference) | 1 | 1 (reference) | 1 |
|  | T2 | 1.48 (0.72-3.04) | 0.284 | 1.37 (0.62-3.03) | 0.441 |
|  | T3 | 1.35 (0.56-3.25) | 0.51 | 1.20 (0.45-3.25) | 0.715 |
|  | T4 | 4.71 (1.91-11.65) | 0.001 | 2.73 (1.00-7.49) | 0.051 |
| N stage | N0 | 1 (reference) | 1 | 1 (reference) | 1 |
|  | N1 | 1.77 (0.93-3.36) | 0.082 | 1.53 (0.76-3.08) | 0.233 |
|  | N2 | 2.81 (1.21-6.53) | 0.016 | 2.62 (1.02-6.75) | 0.046 |
|  | N3 | 4.20 (1.75-10.09) | 0.001 | 4.28 (1.52-12.08) | 0.006 |
| M satge | M0 | 1 (reference) | 1 | 1 (reference) | 1 |
|  | M1 | 11.06 (3.77-32.40) | <0.001 | 5.05 (1.54-16.57) | 0.008 |
|  | Unknown | 1.92 (0.89-4.11) | 0.095 | 1.03 (0.41-2.58) | 0.951 |
| Risk score | Low | 1 (reference) | 1 | 1 (reference) | 1 |
|  | High | 0.52 (0.31-0.89) | 0.017 | 0.56 (0.31-0.85) | 0.046 |
| ER | Negative | 1 (reference) | 1 |  |  |
|  | Positive | 0.88 (0.49-1.60) | 0.684 |  |  |
|  | Unknown | 1.87 (0.53-6.64) | 0.331 |  |  |
| PR | Negative | 1 (reference) | 1 |  |  |
|  | Positive | 0.69 (0.40-1.18) | 0.17 |  |  |
|  | Unknown | 1.46 (0.43-5.00) | 0.543 |  |  |
| HER2 | Negative | 1 (reference) | 1 |  |  |
|  | Positive | 1.26 (0.60-2.68) | 0.543 |  |  |
|  | Unknown | 1.21 (0.67-2.20) | 0.528 |  |  |
| Molecular subtype | Luminal A | 1 (reference) | 1 | 1 (reference) | 1 |
|  | Luminal B | 0.88 (0.54-1.72) | 0.118 | 0.52 (0.36-1.84) | 0.263 |
|  | Her2 enriched | 1.16 (0.85-1.58) | 0.365 | 1.32 (0.50-3.65) | 0.661 |
|  | TNBC | 3.20 (1.66-6.16) | < 0.001 | 2.78 (1.36-5.30) | < 0.001 |
|  | Unknown | 1.28 (0.69-2.75) | 0.373 | 0.87 (0.52-1.92) | 0.525 |
